# Biomarkers of sepsis associated encephalopathy: a bibliometric and visualized analysis

**DOI:** 10.3389/fneur.2025.1605351

**Published:** 2025-06-26

**Authors:** Chuyao Qi, Yanfei Liu, Tianfeng Hua, Min Yang, Yue Liu

**Affiliations:** ^1^Laboratory of Cardiopulmonary Resuscitation and Critical Care, The Second Affiliated Hospital of Anhui Medical University, Hefei, China; ^2^National Clinical Research Center for TCM Cardiology, Xiyuan Hospital of China Academy of Chinese Medical Sciences, Beijing, China

**Keywords:** sepsis associated encephalopathy, biomarkers, bibliometric analysis, CiteSpace, VOSviewer

## Abstract

**Objectives:**

This study employs bibliometric analysis to investigate the current states and emerging trends in the field of sepsis associated encephalopathy biomarkers. It conducts a comparative analysis of the research contributions from different countries, institutions, journals and authors, thereby providing a valuable reference for future investigations in this field.

**Methods:**

All publications on sepsis associated encephalopathy biomarkers research were retrieved and extracted from the China National Knowledge Infrastructure Database and the Web of Science Core Collection on December 31st, 2024. Microsoft Office Excel was used to conduct quantitative analysis of related studies data. VOSviewer, CiteSpace and R package “bibliometrix” were used to conduct the bibliometric analysis.

**Results:**

This study included 248 articles from 36 countries, with China and the United States identified as the primary contributors. The number of publications concerning sepsis associated encephalopathy biomarkers has been progressively rising on an annual basis. Santa Catarina State University, University of Texas Health Science Center at Houston and University of Texas System are the primary research institutions. The largest number of publications appeared in Molecular Neurobiology. Critical Care Medicine is the most co-cited journal. These publications contributed by 1,234 authors among which Felipe Dal-pizzol, Tatiana Barichello and Fabricia Petronilho had published numerous articles and Felipe Dal-pizzol was the most frequently co-cited. “Neuron specific enolase,” “protein” and “oxidative damage markers” are the primary keywords of emerging research hotspots.

**Conclusion:**

This is the first thorough bibliometric study to summarize the developments and trends of sepsis associated encephalopathy biomarkers research since the inception of the China National Knowledge Infrastructure Database and the Web of Science Core Collection. These findings identify recent research hotspots, which will provide a reference for scholars studying sepsis associated encephalopathy biomarkers in the future.

## 1 Introduction

Sepsis is a life-threatening organ dysfunction caused by a dysregulated host response to infection and the global incidence rate is experiencing an upward trend. Sepsis leads to a high mortality rate among patients in the intensive care units and represents one of the major clinical challenges within the field of critical care medicine (1). Sepsis associated encephalopathy (SAE) is a diffuse cerebral dysfunction resulting form sepsis, with clinical manifestations such as alterations in mental state, cognitive deterioration and delirium (2). A research indicates that approximately 53% of sepsis patients will experience SAE (3). In recent years, there has been a significant amount of researches conducted on SAE (4, 5). The comprehensive pathophysiology of SAE remains inadequately understood (6). Nonetheless, various mechanisms have been suggested. SAE appears to be associated with mitochondrial dysfunction, dysfunction of the blood-brain barrier (BBB), oxidative stress, and inflammation (7–9). Clinical assessment tools, including the Glasgow Coma Scale, the Confusion Assessment Method for the Intensive Care Unit, and the Assessment to Intensive Care Environment, are valuable for diagnosing SAE and can function as clinical biomarkers for evaluating SAE prognosis. However, it is important to note that these clinical cores are not applicable to patients who are receiving sedation in the intensive care unit. Therefore, the development of diagnostic instruments, prognostic evaluation, and therapeutic approaches for SAE patients necessitates straightforward, sensitive, and specific predictors and biomarkers (10). Numerous serum biomarkers exhibit elevated levels in SAE patients. Among these biomarkers are procalcitonin, interleukin-8, neuron-specific enolase (NSE), and S100-β protein (11, 12). Therefore, researchers propose that biomarkers may enhance the understanding of the pathological mechanisms involved in SAE and serve as valuable tools for supplementary diagnosis and prognostic assessment of SAE (13–15). Consequently, it is of considerable importance to analyze the current research states and research hotspots in the field of SAE biomarkers, to forecast future development trends and to offer critical insights for the early diagnosis, monitoring, management, and prognostic assessment of SAE. This study applies three bibliometric tools—CiteSpace (16), VOSviewer (17) and R package “bibliometrix” (18)—to analyze the publications concerning SAE biomarkers found within the China National Knowledge Infrastructure Database (CNKI) and the Web of Science Core Collection (WOSCC). The objective is to provide critical guidance for subsequent scholarly research on the diagnosis and prognostic evaluation of SAE.

## 2 Materials and methods

### 2.1 Data sources

Data was retrieved and extracted from the CNKI and the WOSCC on December 31st, 2024. The retrieval time extended from the inception of the databases until December 31st, 2024. The following were the reasons for integrating the WOSCC dataset and the CNKI database into bibliometrics: Firstly, the WOSCC database is recognized as one of the largest and most utilized English-language databases globally, making it a suitable resource for conducting international bibliometric analysis (19). Conversely, the CNKI database stands as one of the largest and most significant repositories of Chinese-language literature, encompassing over 99% of academic journals in China (20). Secondly, the fields of biomarkers and SAE are multidisciplinary, including areas such as medicine, biology and pharmacology. The amalgamation of both Chinese-language literature database and English-language database allows for a more comprehensive exploration of the relationship between biomarkers and SAE.

### 2.2 Search strategy

**Figure 1:**
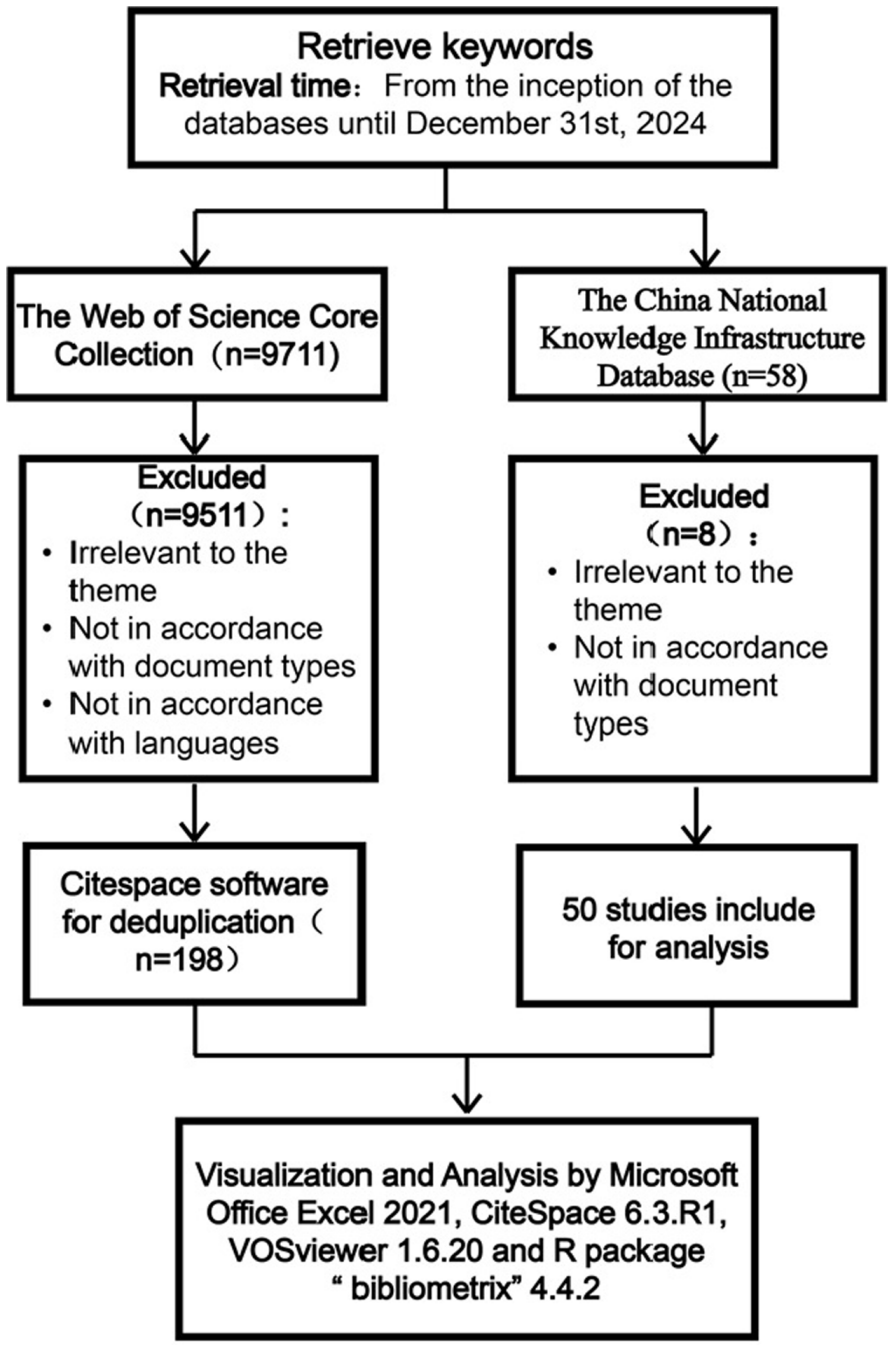
Publications screening flowchart.

The Chinese search terms were sepsis associated encephalopathy, sepsis encephalopathy, septic encephalopathy, septicemic encephalitis, sepsis related delirium, sepsis related brain dysfunction, brain dysfunction caused by sepsis, neurological complications of sepsis, sepsis related acute brain dysfunction, neuroinflammation in sepsis; biomarkers, biomarker, molecular markers, molecular marker, marker, markers. The English search terms were sepsis associated encephalopathy, SAE, sepsis encephalopathy, SE, sepsis-associated delirium, SAD, sepsis-associated brain dysfunction, SABD, sepsis-induced brain dysfunction, sepsis with clinical central nervous system involvement, sepsis-associated acute brain dysfunction, neuroinflammation in sepsis, septic encephalopathy, septic encephalitis; biomarkers, biomarker, biological labeling, biological mark, molecular markers, markers, marker. The search formula in the CNKI was: (SU: sepsis associated encephalopathy + sepsis encephalopathy + septic encephalopathy + septicemic encephalitis + sepsis related delirium + sepsis related brain dysfunction + brain dysfunction caused by sepsis + neurological complications of sepsis + sepsis related acute brain dysfunction + neuroinflammation in sepsis) AND (SU: biomarkers + biomarker + molecular markers + molecular marker + marker + markers). These articles were written in Chinese. The search formula in the WOSCC was: TS = (sepsis associated encephalopathy or (SAE) or (sepsis encephalopathy) or (SE) or (sepsis-associated delirium) or (SAD) or (sepsis-associated brain dysfunction) or (SABD) or (sepsis-induced brain dysfunction) or (sepsis with clinical central nervous system involvement) or (sepsis-associated acute brain dysfunction) or (neuroinflammation in sepsis) or (septic encephalopathy) or (septic encephalitis)) AND TS = (biomarkers or (biomarker) or (biological labeling) or (biological mark) or (molecular markers) or (markers) or (marker)). These articles were written in English. The particular flowchart is shown in Figure 1.

### 2.3 The inclusion criteria

Publications concerning SAE biomarkers that appeared in formally published journals (domestic and international), as well as master’s and doctoral dissertations. The abbreviations present in the keywords utilized for retrieval are either identical to or synonymous with the theme of sepsis associated encephalopathy discussed in this article. The type of documents is designated as “academic papers” and “theses” in the CNKI. The documents types are set to “original articles” and “reviews” in the WOSCC.

### 2.4 The exclusion criteria

The types of documents are meeting abstracts, editorial materials, letters non-peer reviewed sources and others. The abbreviation SAE presents in the keywords utilized for retrieval refers to serious adverse events. The abbreviation SE presents in the keywords utilized for retrieval represents selenium. The abbreviation SAD presents in the keywords utilized for retrieval stands for social anxiety disorder. Duplicate content with previously included references.

### 2.5 Data analysis

This study used VOSviewer (version 1.6.20), CiteSpace (version 6.3.R1) and R package “bibliometrix” (version 4.4.2) for visual analysis. VOSviewer (version 1.6.20) is an advanced bibliometric analysis tool that is proficient in extracting essential information from a diverse range of publications (17). It is commonly employed for the establishment of collaboration, co-citation, and co-occurrence networks. VOSviewer (version 1.6.20) was utilized to analyze countries, institutions, journals, co-cited journals, authors, co-cited authors and keyword co-occurrence visually and conduct cluster analysis in this study. In the map generated by VOSviewer, a node signifies an item, which may include country, institution, journal, or author. The dimensions and coloration of the nodes, respectively, correspond to the quantity and categorization of these items. Additionally, the lines between the nodes signified relationships such as co-authorship or co-citation and the thickness of the lines connecting the nodes represents the collaborative intensity or co-citation frequency among the items (21, 22).

CiteSpace identifies significant or high-impact publications by utilizing various metrics, including citation frequency, centrality, and burst strength. This facilitates researchers in efficiently identifying essential literature within a specific research field (23, 24). We employed CiteSpace (version 6.3.R1) to analyze parameters, such as countries, institutions, authors, journals, topics and references, in addition to performing burst strength.

R package “bibliometrix” (version 4.4.2) was utilized to conduct a comprehensive analysis of collaboration among countries and regions, perform citation analysis of publications, and identify emerging trends in thematic research (25). R package “bibliometrix” (version 4.4.2) was used to analyze collaboration among countries and generate a trend analysis chart depicting the evolution of themes. Additionally, Microsoft Office Excel 2021 was utilized for the quantitative analysis of publications.

## 3 Results

### 3.1 Quantitative analysis of publications

According to the retrieval strategy, there were 58 studies of SAE biomarkers in the CNKI and 9,711 studies of SAE biomarkers in the WOSCC. Employing the aforementioned exclusion criteria, a total of 248 studies, including 50 studies sourced from the CNKI and 198 studies obtained from the WOSCC were selected for bibliometric analysis.

**Figure 2:**
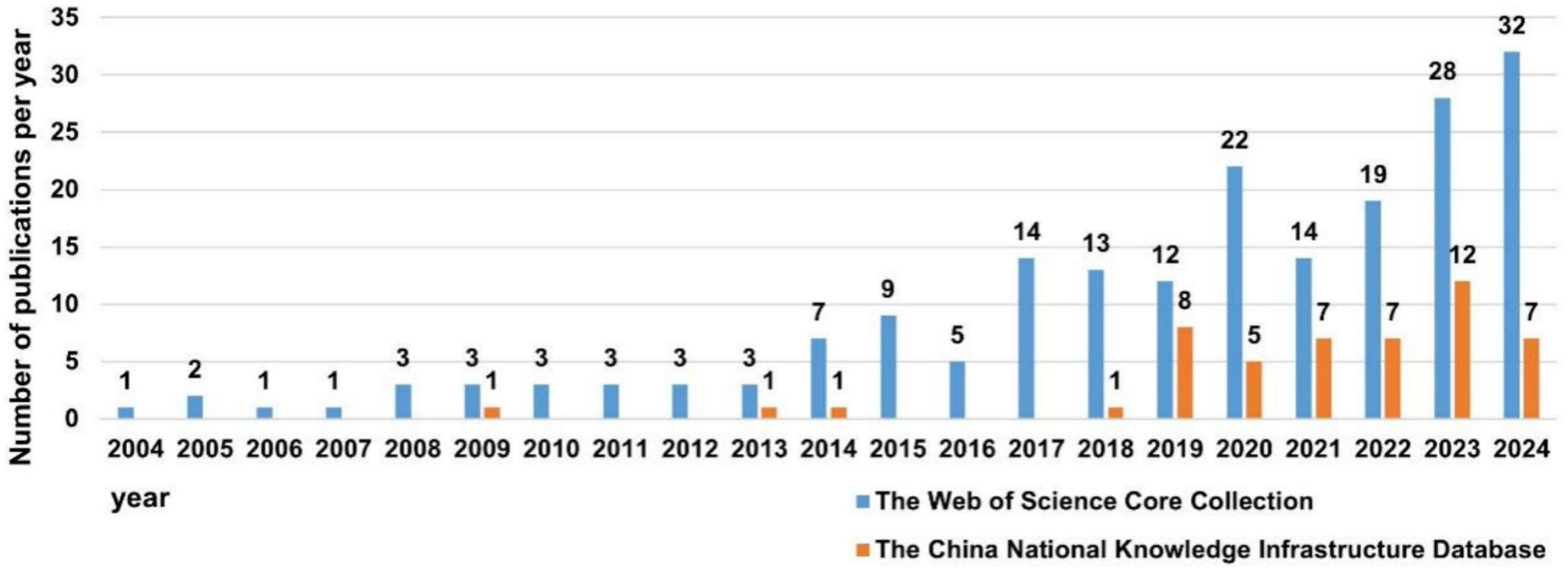
The number of annual publications on SAE biomarkers research.

Based on the annual growth rate of the number of publications in the CNKI, the entire period can be segmented into three distinct parts: Phase 1 (2004–2008), Phase 2 (2009–2019) and Phase 3 (2020–2024). As illustrated in Figure 2, there had been no publications in Phase 1, and the research concerning SAE biomarkers had not been conducted. The number of publications in Phase 2 was comparatively limited, averaging 1.1 articles per year, which was in its nascent stages of SAE biomarkers research. The number of publications in Phase 3 experienced a notable increase, averaging approximately 7.6 articles each year. Overall, over the past 5 years (Phase 3), there has shown an upward trend annually, and the total number of publications during this phase has significantly surpassed that of the other two phases.

As demonstrated in Figure 2, the overall publication trend in the WOSCC can be divided into two parts: Phase 1 (2004–2013) and Phase 2 (2014–2024). The number of publications in Phase 1 was relatively small, averaging 2.3 articles per year, which was in the initial phase of SAE biomarkers research. The number of publications in Phase 2 commenced a notable increase, averaging 15.9 articles per year. In general, over the preceding decade (Phase 2), the number of publications of SAE biomarkers has commenced a marked upward trend year by year, and the total number of publications in this period has experienced a substantial increase in comparison to the other two phases.

Judging from the number of publications, there has been a notable increase in both domestic and international articles concerning SAE biomarkers from 2020 to 2024, with a predominance of English-language publications over those in Chinese.

### 3.2 Country and institutional analysis

**Figure 3:**
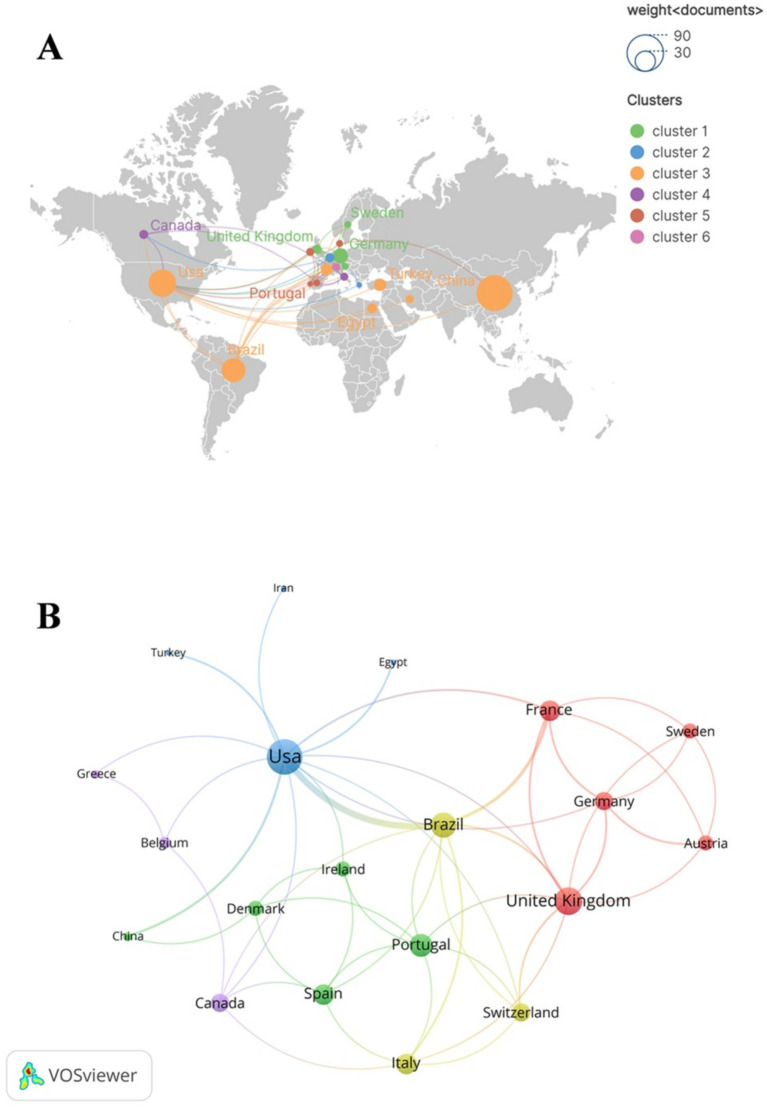
The geographical distribution **(A)** and country visualization **(B)** of SAE biomarkers research in the WOSCC. In the geographical distribution **(A)**, each node represents a specific country or region, with the dimensions of the nodes reflecting the quantity of publications from these countries or regions. The thickness of the lines that connect the nodes indicates the extent of collaborative interactions among the countries or regions. Additionally, the color of each node is associated with the cluster classification presented on the right side of the figure.

These publications were co-authored by scholars from 36 countries. Among these countries, China has the highest number of publications (*n* = 79), followed by The United States (*n* = 46), Brazil (*n* = 35). In co-authorship networks, nodes exhibiting high centrality (institutions or countries), typically occupy a central position within the collaborative framework. These nodes (institutions or countries) possess significant influence and play a pivotal role in the dissemination of knowledge (26). The United States (0.59), Brazil (0.15), China (0.12) and England (0.11) have relatively high centrality, as demonstrated in Table 1, suggesting that they possess substantial innovation capabilities and exert considerable influence in the field of SAE biomarkers research. Subsequently, we conducted a filtration and visualization process involving 36 countries based on a minimum of two publications. Then, we established a collaborative network that reflected both the quantity and relationship of publications across these countries, as illustrated in Figure 3. It is noteworthy that there exists significant collaboration among various countries. For instance, China has active relationships with the United States and Denmark, while the United States engages in close cooperation with Brazil, Germany and France.

**Table 1:** Top 10 countries in SAE biomarkers research.

Rank	Count	Centrality	Country
1	79	0.12	China
2	46	0.59	USA (The United States)
3	35	0.15	Brazil
4	14	0.03	Germany
5	9	0	Turkey
6	8	0.04	France
7	6	0	Egypt
8	5	0	Belgium
9	5	0.03	Canada
10	5	0.11	England

**Figure 4:**
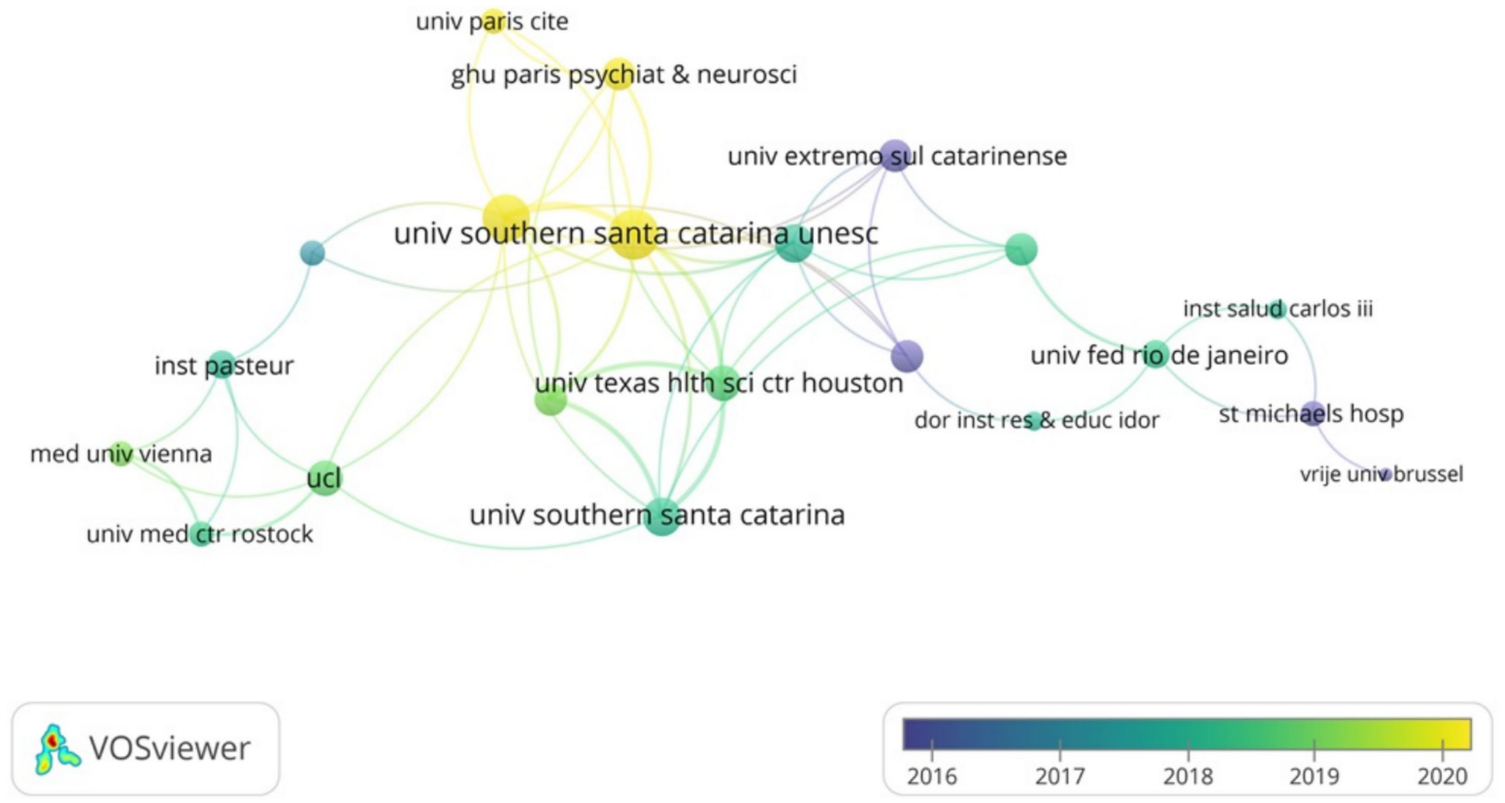
The visualization of institutions of SAE biomarkers research in the WOSCC.

As shown in Table 2, the institution with the highest number of publications in the WOSCC is Santa Catarina State University (*n* = 16), followed by University of Texas Health Science Center at Houston (*n* = 14), University of Texas System (*n* = 14) and others. Subsequently, we filtered and visualized institutions based on the criterion of having published a minimum of two articles, constructing a collaborative network (Figure 4) based on the number of publications and interconnections among the institutions. As illustrated in Figure 4, there exists a strong collaborative relationship among Santa Catarina State University and University of Texas Health Science Center at Houston. Additionally, there is active collaboration among University College London, University Medical Centre Rostock and Pasteur Institute.

**Table 2:** Top 10 institutions in SAE biomarkers research in the WOSCC.

Rank	Count	Institution
1	16	Santa Catarina State University
2	14	University of Texas Health Science Center at Houston
3	14	University of Texas System
4	13	Baylor College of Medicine
5	9	Nanjing Medical University
6	8	Central South University
7	6	Southeast University
8	6	Istanbul University
9	6	Egyptian Knowledge Bank
10	6	Nanjing University

As demonstrated in Table 3, Central South University published the highest number of articles (*n* = 4) in the CNKI, followed by Chongqing Medical University (*n* = 3) and Air Force Medical University (*n* = 2).

**Table 3:** Top 10 institutions in SAE biomarkers research in the CNKI.

Rank	Count	Institution
1	4	Central South University
2	3	Chongqing Medical University
3	2	Air Force Medical University
4	2	Huazhong University of Science and Technology
5	2	Dalian Medical University
6	2	Peking Union Medical College
7	2	Lanzhou University
8	1	Nanjing University
9	1	Pediatric Intensive Care Unit in Shenzhen Bao’an Women’s and Children’s Hospital
10	1	Emergency Medicine Department of Lanzhou University Second Hospital

### 3.3 Journals and co-cited journals

**Figure 5:**
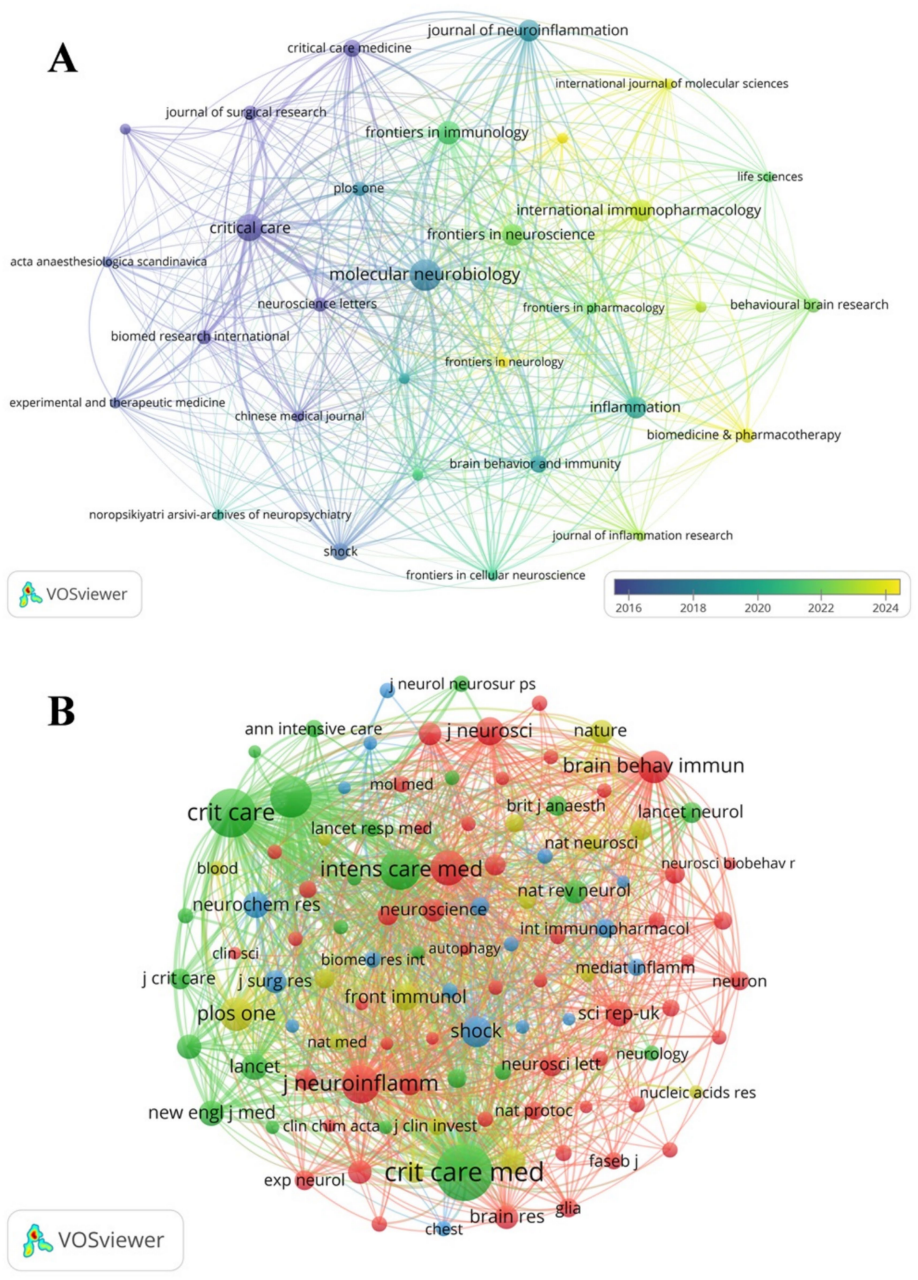
The visualization of SAE biomarkers research journals **(A)** and co-cited journals **(B)** in the WOSCC.

The studies of SAE biomarkers in the WOSCC were published in 117 journals. As illustrated in Table 4, Molecular Neurobiology published the most articles (*n* = 12), followed by Critical Care (*n* = 9) and Frontiers in Immunology (*n* = 7). Among the top 10 journals, Journal of Neuroinflammation holds the highest impact factor (IF = 9.3), followed by Critical Care (IF = 8.8) and Brain Behavior and Immunity (IF = 8.8). Subsequently, we conducted a screening of journals based on the criterion of a minimum of two pertinent publications and created the journal network (Figure 5A). As shown in Figure 5A, Molecular Neurobiology maintains active citation relationships with Critical Care, Frontiers in Immunology and Brain Behavior and Immunity.

**Table 4:** Top 10 journals in SAE biomarkers research in the WOSCC.

Rank	Journal	Count	Citation	IF (2023)
1	Molecular Neurobiology	12	350	4.6
2	Critical Care	9	537	8.8
3	Frontiers in Immunology	7	170	5.7
4	Inflammation	6	107	4.5
5	Frontiers in Neuroscience	6	45	3.2
6	Journal of Neuroinflammation	6	1,015	9.3
7	International Immunopharmacology	6	23	4.8
8	Brain Behavior and Immunity	4	306	8.8
9	Shock	4	158	2.7
10	Critical Care Medicine	4	258	7.7

Among the top 10 co-cited journals, one journal was cited more than 500 times. Critical Care Medicine (co-citation = 537) was the most cited journal. We filtered journals based on a minimum of 20 co-citations and constructed the co-citation network (Figure 5B). As demonstrated in Figure 5B, Critical Care Medicine has close citation relationships with Critical Care, Intensive Care Medicine and JAMA-Journal of the American Medical Association.

### 3.4 Authors and co-cited authors

**Figure 6:**
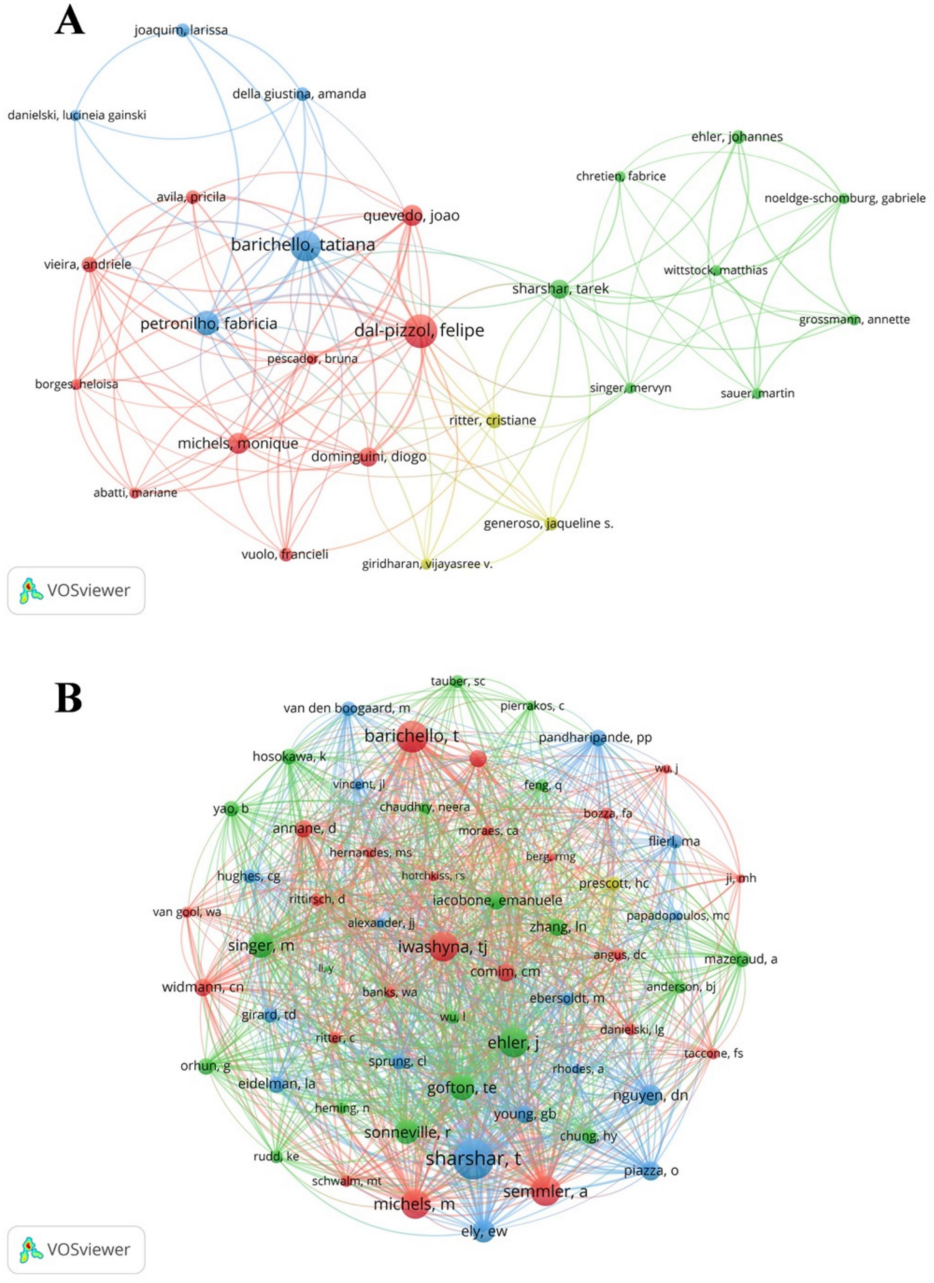
The visualization of SAE biomarkers research authors **(A)** and co-cited authors **(B)** in the WOSCC.

A total of 1,234 authors contributed to SAE biomarkers research in the WOSCC. Among the top 10 authors, three authors published 10 or more articles (Table 5). We established a collaborative network comprising authors who had published three or more articles, as illustrated in Figure 6A. The nodes for Felipe Dal-pizzol, Tatiana Barichello and Fabricia Petronilho are distinguished by their prolific output. Furthermore, we noted close collaboration among various authors. For instance, Felipe Dal-pizzol had close cooperation with Tatiana Barichello, Fabricia Petronilho and others, while Tatiana Barichello maintained positive relationships with Joao Quevedo, Monigue Michels and others.

**Table 5:** Top 10 authors in SAE biomarkers research in the WOSCC.

Rank	Author	Count	Citation
1	Felipe Dal-pizzol	18	866
2	Tatiana Barichello	16	553
3	Fabricia Petronilho	11	429
4	Joao Quevedo	8	457
5	Monigue Michels	8	408
6	Diogo Dominguini	7	274
7	Tarek Sharshar	7	406
8	Andriele Vieira	5	149
9	Cristiane Ritter	5	208
10	Figen Esen	5	80

Furthermore, we recorded the total citations of authors. The most cited author is Felipe Dal-pizzol (citation = 866), followed by Tatiana Barichello (citation = 553). We filtered authors with the minimum co-citation count of 15 and mapped the co-citation network (Figure 6B). As demonstrated in Figure 6B, there exists active collaboration among multiple co-cited authors, such as Tarek Sharshar and Tatiana Barichello and Monigue Michels.

### 3.5 Co-cited references

**Figure 7:**
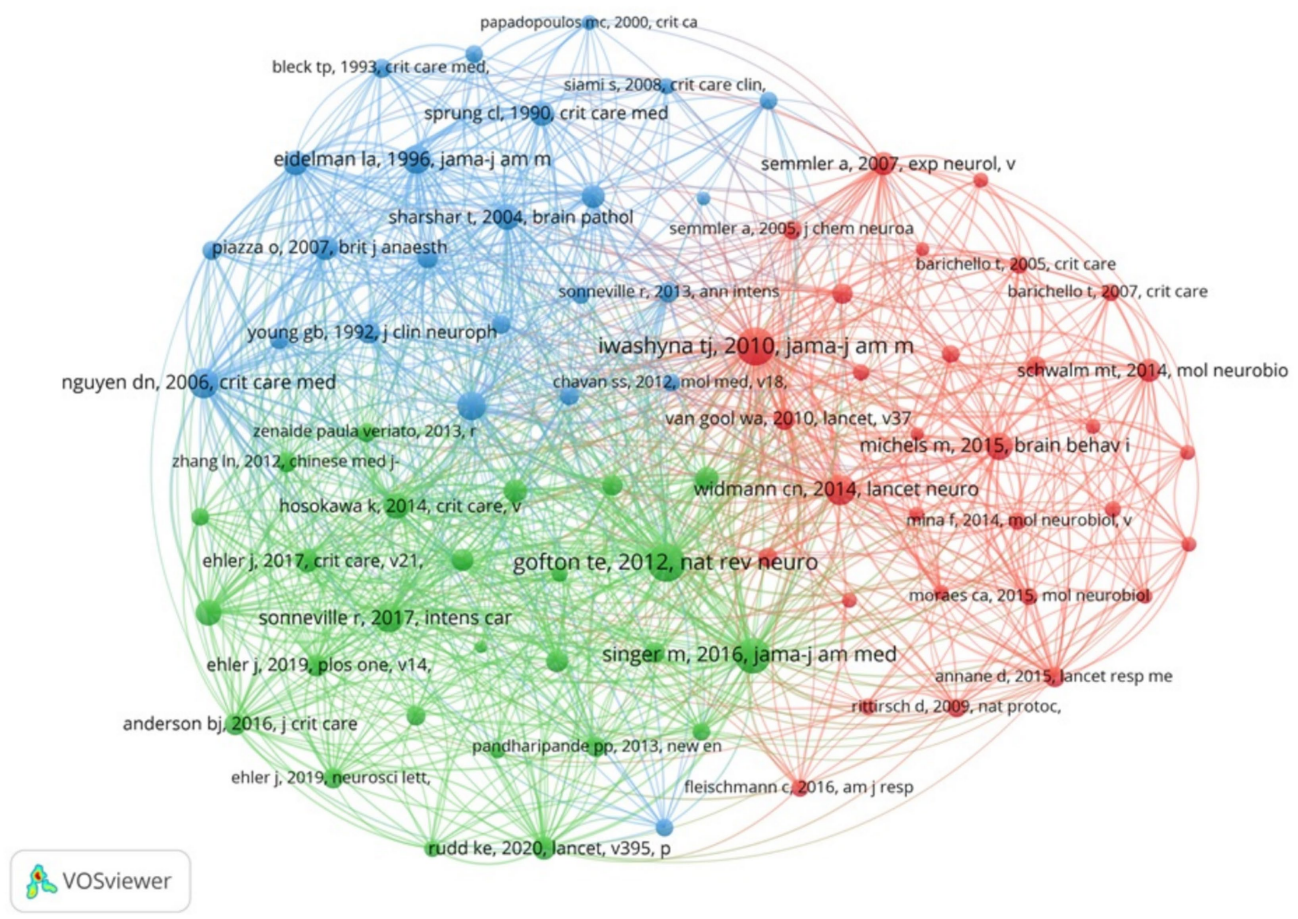
The visualization of co-cited references of SAE biomarkers research in the WOSCC.

There are 8,007 co-cited references concerning SAE biomarkers research in the WOSCC. We constructed the co-citation network (Figure 7) using references that had been co-cited 10 or more times. As shown in Figure 7, “Iwashyna T. J., 2010, JAMA-J Am Med Assoc” shows positive co-citation relationships with “Gofton T. E., 2012, Nat Rev Neurol,” “Singer M., 2016, JAMA-J Am Med Assoc” and “Nguyen D. N., 2006, Crit Care Med.”

### 3.6 Reference with citation bursts

**Figure 8:**
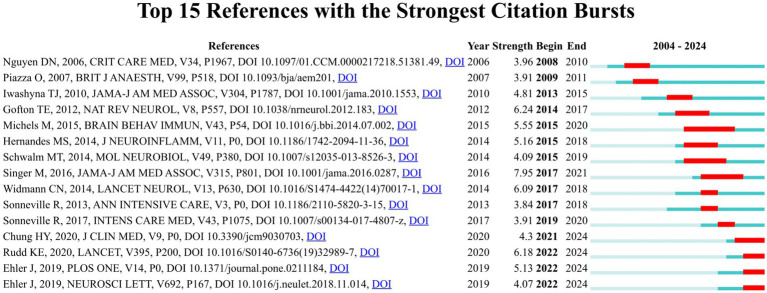
Top 15 references with robust citation bursts in the WOSCC.

References with citation bursts are the references widely cited by researchers within a specific field over a period of time. The citation burst strength is frequently employed as an indicator to identify outbreak points of nascent research trends or high impact scholarly works (27). We employed CiteSpace to identify 15 references with robust citation bursts in the WOSCC. The blue line represents the duration of citations from the inception of the database to 2024, while the red bar represents the citation burst strength (28). As illustrated in Figure 8, citation bursts for references were observed as early as 2008 and as recently as 2022. The article entitled “the third international consensus definitions for Sepsis and septic shock (Sepsis-3)”, authored by Mervyn Singer et al., exhibited the most robust citation burst (intensity = 7.95), with citation bursts from 2017 to 2021. The reference with the second robust citation burst (strength = 6.24) was titled “Sepsis-associated encephalopathy,” published by Teneille E. Gofton et al. with citation bursts from 2014 to 2017. Overall, the citation burst strength of the top 15 references ranged from 3.84 to 7.95. Table 6 summarizes the main research findings from these 15 references.

**Table 6:** Top 15 references with robust citation bursts in the WOSCC.

Rank	Strength	The main research findings
1	3.96	Elevated serum levels of S-100β protein and neuron-specific enolase are associated with brain injury in patients with severe sepsis and septic shock (14)
2	3.91	Elevated S100B levels do not correlate with the severity of encephalopathy during sepsis (72)
3	4.81	Long-term cognitive impairment and functional disability among survivors of severe sepsis (89)
4	6.24	Sepsis-associated encephalopathy (54)
5	5.55	The role of microglia activation in the development of sepsis-induced long-term cognitive impairment (56)
6	5.16	The role of Nox2-derived ROS in the development of cognitive impairment after sepsis (90)
7	4.09	Acute brain inflammation and oxidative damage are related to long-term cognitive deficits and markers of neurodegeneration in sepsis-survivor rats (41)
8	7.95	The third international consensus definitions for sepsis and septic shock (sepsis-3) (1)
9	6.09	Long-term cerebral consequences of sepsis (55)
10	3.84	Understanding brain dysfunction in sepsis (91)
11	3.91	Potentially modifiable factors contributing to sepsis-associated encephalopathy (3)
12	4.3	Sepsis-associated encephalopathy: from delirium to dementia? (92)
13	6.18	Global, regional, and national sepsis incidence and mortality, 1990–2017: analysis for the Global Burden of Disease Study (93)
14	5.13	The prognostic value of neurofilament levels in patients with sepsis-associated encephalopathy—a prospective, pilot observational study (94)
15	4.07	Diagnostic value of NT-proCNP compared to NSE and S100B in cerebrospinal fluid and plasma of patients with sepsis-associated encephalopathy (95)

### 3.7 Hotspots and frontiers

**Figure 9:**
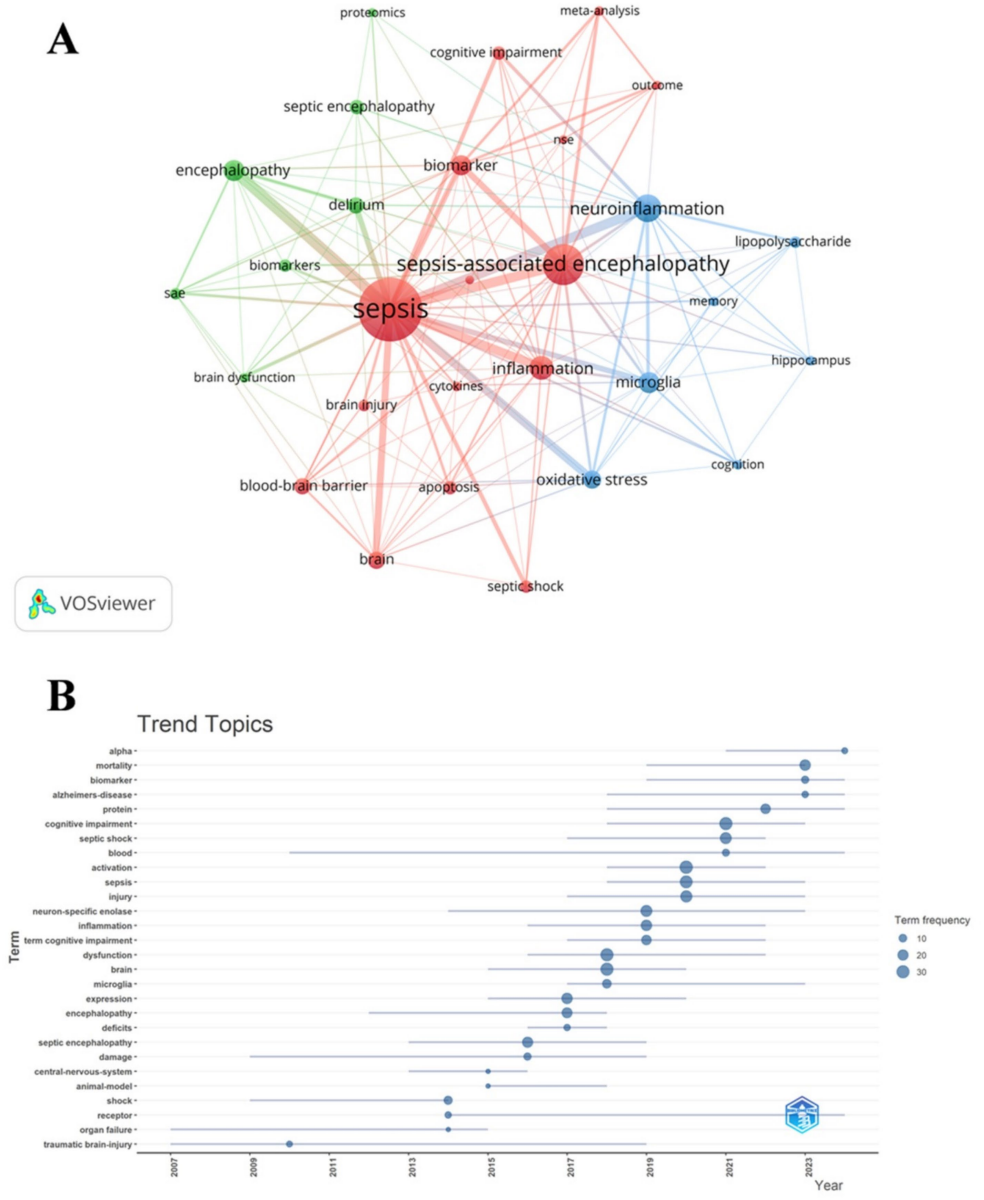
Keyword cluster analysis **(A)** and thematic trend analysis **(B)** in the WOSCC.

We conducted a co-occurrence analysis of keywords to quickly identify emerging research hotspots within a specific field. Table 7 demonstrates the top 20 high-frequency keywords in SAE biomarkers research in the WOSCC. Neuron specific enolase and protein represented the main research directions of SAE biomarkers internationally. Keywords clustering analysis categorizes research topics into clusters by examining the co-occurrence relationships of keywords. This methodology allows for the identification of current hotspots and future development trends within a research field (29). When combined with the citation burst strength, as previously outlined, this approach facilitates the identification of emerging domains, tracking of research hotspots, and analysis of future directions in bibliometric studies. We filtered keywords with the minimum frequency of 4 and conducted a cluster analysis through VOSviewer (Figure 9A). The thickness of the lines between the nodes is indicative of the strength of the relationships between the keywords. As shown in Figure 9A, we identified a total of three clusters, denoting three research directions for future research on SAE biomarkers. The green cluster includes keywords such as biomarkers, delirium, proteomics and others, indicating that some future research may prioritize the identification of SAE biomarkers predominantly associated with proteins. The red cluster comprises terms like apoptosis, biomarker, blood-brain barrier, cytokines, NSE and others, suggesting that some future research may focus primarily on cytokines that have the ability to traverse the BBB. The keywords in blue cluster encompass hippocampus, lipopolysaccharide, microglia and others, indicating that some future research may steer towards the discovery of SAE biomarkers specifically linked to hippocampal and microglial damage. We utilized R package “bibliometrix” to generate a trend analysis chart depicting the evolution of themes (Figure 9B). As illustrated in Figure 9B, the keywords neuron-specific enolase and protein have been frequently cited in the last 5 years (2020–2024), suggesting that they may represent current research focal points regarding SAE biomarkers.

**Table 7:** Top 20 keywords in SAE biomarkers research in the WOSCC.

Rank	Count	Keyword
1	45	sepsis-associated encephalopathy
2	38	cognitive impairment
3	32	activation
4	30	brain
5	30	dysfunction
6	23	sepsis
7	23	injury
8	22	septic shock
9	21	septic encephalopathy
10	20	expression
11	20	neuron specific enolase
12	19	neuroinflammation
13	19	inflammation
14	18	delirium
15	18	oxidative stress
16	16	cecal ligation
17	16	mortality
18	15	encephalopathy
19	14	blood-brain barrier
20	13	protein

Table 8 shows the top 20 high-frequency keywords in SAE biomarkers research in the CNKI. Procalcitonin, cytokine, lipid metabolism, oxidative stress and blood ammonia represented the main locally prevalent research themes of SAE biomarkers.

**Table 8:** Top 20 keywords in SAE biomarkers research in the CNKI.

Rank	Count	Keyword
1	23	sepsis
2	4	inflammation
3	3	prognosis
4	3	diagnosis
5	2	electroencephalogram
6	2	cognitive impairment
7	2	blood brain barrier
8	2	pathogenesis
9	2	cerebral injury
10	2	inflammatory response
11	2	lipopolysaccharide
12	1	procalcitonin
13	1	cytokine
14	1	oxidative stress
15	1	lipid metabolism
16	1	blood ammonia
17	1	Iron death
18	1	mitochondria
19	1	synapse injury
20	1	immune disorders

## 4 Discussion

### 4.1 General information

A total of 248 studies related to SAE biomarkers have been published both domestically and internationally from the inception of the databases to 2024. Since 2004, there has been a gradual emergence of scholarly international articles on SAE biomarkers. An article titled “Time-dependent mitochondrial-mediated programmed neuronal cell death prolongs survival in sepsis* “provides a research direction for SAE biomarkers studies internationally, suggesting that the expression of cell death effector proteins in the brain cells of SAE mice may serve as an indicator of brain damage associated with SAE (30). Until 2013, this field of research was still in the initial phase, characterized by an average annual publication rate of only 2.3 articles. From 2014 and 2024, there was a notable increase in the volume of publications internationally, with an average of 15.9 articles annually. This notable rise in the volume of publications may be attributed to the dissemination of numerous high-impact articles that offer critical insights for clinical trials and the identification of the most effective biomarker combinations for the diagnosis, treatment, and prognostic assessment of patients with SAE (31–33).

From 2004 to 2008, there was a notable absence of publications regarding SAE biomarkers in China, suggesting a lack of research activity in this area during that period. Since 2009, there has been a progressive increase in the publication of academic articles focused on SAE biomarkers within the domestic scholarly community. An article advocating for the beneficial implications of early assessment of serum levels of NSE and S100-β protein in predicting the status and prognosis of SAE offers a valuable research avenue for the study of SAE biomarkers in China (34). Until 2019, this area of research remained in its nascent stages, averaging 1.1 articles per year. From 2020 and 2024, there was a notable increase in the volume of publications domestically, with an average of 7.6 articles annually. The rise in the volume of publications may be attributed to the dissemination of several significant domestic studies that suggest the measurement of biomarkers, such as NSE and S100-β protein in serum, holds considerable value for the early diagnosis and evaluation of the severity of SAE (35–37). The number of domestic and international publications has rapidly increased over the past 5 years, indicating that the research on SAE biomarkers has attracted increasing attention from scholars.

China and the United States are major countries engaged in research on SAE biomarkers. China has close relationships with the United States and Denmark, while the United States maintains active collaboration with Brazil, Germany and France. It is important to highlight that the collaboration between Brazil and the United States is more robust than that between China and the United States. This observation indicates that, as the major countries contributing significantly to the research of SAE biomarkers, China and the United States should engage in more comprehensive cooperation and exchanges in the future to jointly promote the field of SAE biomarker research. Among research institutions, Santa Catarina State University in Brazil, an emerging institution that emerged after 2020, has made the most significant contributions to the research on SAE biomarkers. There exists close collaboration among numerous institutions, such as Santa Catarina State University and University of Texas Health Science Center at Houston. It is noteworthy that there exists a limited degree of collaboration between Chinese institutions and international institutions. Consequently, it is essential for both domestic and international institutions to enhance their cooperation and exchanges in order to collectively advance the field of SAE biomarkers research.

The journal that has published the highest number of articles related to SAE biomarkers research is Molecular Neurobiology (IF = 4.6, Q1), suggesting that the hotspots in this research field in recent years have predominantly centered on molecular-level biomarkers. The journal with the highest impact factor is Journal of Neuroinflammation (IF = 9.3, Q1), followed by Critical Care (IF = 8.8, Q1). The former is a very influential journal in the field of neuroinflammation research, while the latter is a significant publication in the field of critical illness research, including conditions such as sepsis. These highly influential journals are likely to offer valuable resources for future research on SAE biomarkers.

From the standpoint of authorship, Felipe Dal-pizzol, Tatiana Barichello and Fabricia Petronilho published relatively more articles, each contributing over 10 publications. Professor Felipe Dal-pizzol has published 18 related articles, several of which summarize biomarkers applicable for the assessment of SAE, such as NSE (38), S100-β protein (31, 38), brain-derived neurotrophic factor (BDNF) (38–40), amyloid β-protein (Aβ) (41–44) and others. Tatiana Barichello and Felipe Dal-pizzol collaboratively authored a total of 13 articles, the majority of which focused on the identification of inflammatory cytokines in brain tissue as diagnostic and prognostic biomarkers for SAE (45–49). Fabricia Petronilho and Tatiana Barichello jointly published three articles that utilized oxidative damage markers in brain tissue, including thiobarbituric acid reactive substances (TBARS) and protein carbonyl content, to evaluate SAE injury (50–52). Overall, contemporary researches on SAE biomarkers predominantly concentrate on inflammatory cytokines, NSE, S100-β protein and oxidative damage markers present in brain tissue.

### 4.2 Knowledge base

Co-cited references pertain to those references cited by multiple other scholarly works, which can assist researchers in gaining a deeper comprehension of the dynamics within a particular research domain, identifying seminal literature, and uncovering previously overlooked research avenues (53). We identified the four high co-citation counts references to understand the research evolution of SAE biomarkers. In 2006, Nguyen et al. (14) published one article in Critical Care Medicine, demonstrating that serum levels of NSE and S100-β protein can serve as indicators for assessing the extent of brain injury in SAE patients and can also be utilized to predict their prognosis. The most co-cited research, published by Gofton and Young (54) established in 2012, introduced several SAE biomarkers, including S100-β protein and NSE. However, scholars noted the absence of specific biomarkers for SAE during that period, indicating that the clinical diagnosis of SAE primarily relied on the exclusion of primary central nervous system infections and other etiologies of encephalopathy. Currently, S100-β protein and NSE remain the research hotspots of SAE biomarkers. Among the four high co-citation counts articles, Widmann (55) published an article in The Lancet Neurology in 2014. This research posited that inflammatory cytokines, including interleukin-6 (IL-6), interleukin-1β (IL-1β) and tumor necrosis factor-α (TNF-α), can be identified in brain tissue following SAE. A study published by Michels et al. (56) in 2015 identified various oxidative damage markers associated with SAE damage, such as TBARS, protein carbonyl content and others. In summary, the references that receive high co-citation counts primarily focus on the following topics: NSE, S100-β protein, inflammatory cytokines and oxidative damage markers, which serve as theoretical foundation for subsequent SAE biomarkers research.

### 4.3 Hotspots and frontiers

References with citation bursts indicate the emerging themes within a specific research field (57). According to the main research findings of references with robust citation bursts (Table 5), we can find that S100-β protein, NSE, inflammatory cytokines and oxidative damage markers are the hotspots in the field of SAE biomarkers.

Additionally, the utilization of keywords can facilitate a rapid understanding of the research hotspots and developments within the domain of SAE biomarkers. Table 7 primarily encompasses the following keywords internationally: NSE, protein and oxidative stress. Meanwhile, Table 8 illustrates that the primary keywords in the domestic research domain encompass the following: cytokines, lipid metabolism and oxidative stress. In summary, our analysis reveals that both domestic and international researchers in the field of SAE biomarkers have recognized the significance of oxidative stress-related biomarkers. However, while international studies predominantly concentrate on biomarkers centered around NSE and proteins, Chinese scholars have additionally explored the application of biomarkers associated with lipid metabolism and cytokines in SAE biomarkers research. Currently, there exists a limited number of pertinent studies, with only a single publication available for each category. This lack of substantial evidence hinders the establishment of a comprehensive theoretical framework for clinical application. Consequently, systematic and rigorous investigations along these research directions remain imperative to facilitate progress in the SAE biomarkers field. Our analysis primarily focused on keywords derived from the WOSCC, which are considered to be the prominent hotspots within the research domain of SAE biomarkers due to the lack of domestic retrieval data. According to keyword cluster analysis and thematic trend analysis (Figure 9), the research of SAE biomarkers mainly concentrates on the following aspects.

#### 4.3.1 Neuron specific enolase

Through in-depth study on SAE, researchers discovered that utilizing serum biomarkers to evaluate the extent of brain injury associated with SAE is more convenient than employing more complex diagnostic methods. These methods include the Glasgow Coma Scale upon admission, somatosensory evoked potentials, electroencephalography and imaging technologies (14), and the analysis of plasma amino acids such as tyrosine and phenylalanine (58). NSE serves as a significant serum biomarker for brain injury, as it is a glycolytic enzyme predominantly found in neurons and neuroendocrine cells (59). Furthermore, researches have indicated that the concentrations of NSE in serum and cerebrospinal fluid increase in head trauma (60) and stroke (61), so NSE seems significant as a biomarker to assess brain injury in SAE patients. Nguyen et al. (14) posited that there is a correlation between brain injury in patients suffering from sepsis and elevated serum levels of NSE.

Significant developments have been achieved in the investigation of NSE as a biomarker for the supplementary diagnosis and evaluation of brain injury in SAE patients. However, the specificity of NSE in diagnosing and evaluating brain injury within this patient population remains a subject of debate. Certain researchers posited that elevated serum levels of NSE may be associated with sepsis and other severe medical conditions, or may be indicative of mild brain injury resulting from inflammatory processes. This relationship has yet to be definitively established (62). Additionally, the levels of NSE are affected by multiple factors, including age, sex and muscle injury (63). Finally, NSE is present in red blood cells and platelets, so hemolysis may result in elevated serum NSE levels. This phenomenon is likely to compromise the accuracy of NSE as a biomarker for the diagnosis and evaluation of SAE (64). Consequently, it is imperative to take into account a range of non-neurological disease factors in its clinical application when utilizing NSE as a biomarker for the diagnosis and evaluation of SAE-associated brain injury.

In conclusion, NSE is insufficient as the diagnostic criteria for SAE-associated brain injury, as it cannot substitute for the clinical manifestations of SAE or other diagnostic assessments. A comprehensive analysis that incorporates other examination indicators and the clinical manifestations of SAE is essential for accurate evaluation. Therefore, the assessment of serum NSE levels should be integrated with the evaluation of additional biomarkers, including S100-β protein and inflammatory cytokines, and relevant diagnostic techniques such as electroencephalography, computed tomography and magnetic resonance imaging and others. This comprehensive approach is essential for enhancing the diagnostic accuracy of SAE, assessing patients prognosis, and informing treatment strategies.

#### 4.3.2 Protein

Currently, there are multiple diagnostic approaches for SAE, including the assessment of clinical manifestations in patients, biochemical tests, neuroimaging examinations, ultrasound evaluations, electroencephalography and others (65). Nonetheless, these diagnostic approaches present several drawbacks, including invasiveness, insufficient specificity and the necessity for high-cost devices. In contrast, measuring biomarkers levels in serum or cerebrospinal fluid is more simplified and can be implemented as a routine examination for SAE patients (66). Numerous studies have been conducted on SAE biomarkers, including S100-β protein, inflammatory cytokines, BDNF, Aβ and other proteins.

S100-β protein, also known as S100B protein, is a calcium-binding protein predominantly found in astrocytes and oligodendrocytes within the central nervous system, as well as in Schwann cells of the peripheral nervous system (15). Under normal physiological conditions, S100-β is predominantly located in the cytoplasm. However, under pathological conditions, it is secreted by astrocytes. The varying concentrations of it yield distinct biological effects: at nanomolar concentrations, it facilitates the growth of neuronal axons, while at micromolar concentrations, it induces apoptosis in both astrocytes and neurons (67). S100-β protein serves as a biomarker for evaluating the severity of brain injury in various neurological conditions, including traumatic brain injury without multiple trauma (68), acute stroke (69) and Alzheimer’s disease (70) and others. Erikson et al. (71) discovered that elevated serum levels of S100-β protein in patients experiencing septic shock were associated with the occurrence of delirium. The results indicate that S100-β protein has the potential to serve as a biomarker for the evaluation and diagnosis of brain injury in SAE patients. Nonetheless, the inconsistency in research findings has led to ongoing debate regarding the utility of S100-β protein in the screening and monitoring of SAE. Piazza et al. (72) reported that the elevation of serum S100-β protein levels did not correlate with the severity of brain injury in SAE patients. However, the small sample size of this study raises questions regarding the efficacy of S100-β protein as a prognostic indicator for brain injury in SAE patients. A research demonstrated a significant correlation between serum S100-β protein levels and the severity of brain injury in SAE patients. While S100-β protein has proven to be both effective and sensitive for diagnosing SAE, its specificity remains limited (15). Consequently, subsequent researches should utilize a sufficiently large sample size of SAE patients and animal models to investigate the specific mechanisms underlying the elevated levels of S100-β protein in serum in relation to the onset of brain injury associated with SAE. This will facilitate the assessment of whether S100-β protein can be integrated with other diagnostic approaches, such as clinical manifestations and electroencephalography, to enhance the diagnosis and evaluation of brain injury in individuals with SAE.

Neuroinflammation, oxidative stress, and the BBB dysfunction are the primary mechanisms underlying the pathogenesis of SAE (73). Systemic inflammation and oxidative stress contribute to the loss of the BBB integrity, facilitating the entry of peripheral inflammatory mediators into the central nervous system (74). Upon the loss of the BBB, resting microglia are swiftly activated and subsequently secrete various inflammatory cytokines, including TNF-α, IL-6, and IL-1β (75). A study indicated that the concentrations of IL-6 and interleukin-8 in the cerebrospinal fluid of patients diagnosed with SAE were elevated (76). Additionally, scholars identified elevated concentrations of inflammatory cytokines, including IL-1β and TNF-α, within the brain tissue of septic mice (77). These findings indicate that the concentrations of inflammatory cytokines present in brain tissue or cerebrospinal fluid could serve as potential biomarkers to aid in the diagnosis and evaluation of SAE-associated brain injury.

BDNF is a neurotrophic protein that is extensively present in the cerebral cortex and hippocampus. It plays a crucial role in enhancing the survival of neurons, as well as facilitating synaptic development and activity (78). A research indicated that the concentrations of BDNF in both the hippocampus and the entire brain of mice diminished 1 month following the administration of lipopolysaccharide (79). These findings suggest that BDNF may become a biomarker associated with brain dysfunction in SAE.

Aβ is a protein generated through the hydrolytic cleavage of amyloid precursor protein by the enzymes β-secretase and γ-secretase (80). In neurodegenerative disorders, including Alzheimer’s disease, the accumulation of Aβ in the extracellular space results in the formation of amyloid plaques, ultimately contributing to neuronal death (81). A study conducted on animals demonstrated that rats with sepsis exhibited cognitive impairments and elevated levels of Aβ in the brain tissue (41). Gasparotto et al. (43) also observed an elevation in the levels of Aβ in the brain tissue of septic rats. However, following the administration of an anti-advanced glycation end product antibodies, there was a notable recovery in the cognitive function of the rats, accompanied by a reduction in the levels of Aβ in the brain tissue. These results indicate that Aβ may serve as a significant biomarker for the diagnosis and evaluation of SAE-associated brain injury.

#### 4.3.3 Oxidative damage markers

The progress of brain injury in the context of sepsis is associated with oxidative damage to lipids, proteins and other substances (82). TBARS is commonly selected as an indicator of lipid peroxidation (83). Meanwhile, protein carbonyl content is quantified to evaluate the impact of oxidative stress on proteins (84). Numerous studies have demonstrated that the concentrations of TBARS and protein carbonyls in the brain tissue of sepsis animal models are increased (50, 52, 56). The findings indicate that the assessment of oxidative damage markers in brain tissue may prove beneficial for the diagnosis and prognostic evaluation of brain injury in SAE patients in the future.

While numerous biomarkers, including NSE, S100-β protein, and TBARs, are presently recognized as indicators of brain injury in individuals with SAE and serve as supplementary instruments for prognostic evaluation. It is important to note that, as previously stated, no single biomarker currently exists that can reliably evaluate the prognosis of brain injury in patients with SAE (85–88). Consequently, subsequent research initiatives should prioritize the identification of multiple biomarkers exhibiting high sensitivity and specificity. By conducting a thorough analysis that incorporates these biomarkers alongside the clinical manifestations of SAE and various diagnostic assessments, including electroencephalography, the Glasgow Coma Scale upon admission, somatosensory evoked potentials and others, we can strive for enhanced accuracy in the diagnosis and prognostic evaluation of patients. This research employed bibliometric methods to perform the first comprehensive visualization analysis of SAE biomarkers research, utilizing three distinct bibliometric tools, facilitating a more comprehensively and in-depth comprehension of the research hotspots and developments in this field for scholars. However, this study exhibits certain limitations: firstly, it exclusively encompassed original articles and reviews, omitting meeting abstracts, editorial materials, letters and other types of references. Nevertheless, during the literature screening process, it was observed that the number of articles outside the categories of original articles and reviews was minimal. Consequently, this exclusion is unlikely to significantly affect the overall development trend of the research domain concerning SAE biomarkers. Secondly, the present study exclusively incorporated articles published in English and indexed in the WOSCC since the inception of the database. This decision was made due to the minimal availability of relevant content in other languages identified during the screening process. Consequently, the restriction to English-language publications has only a marginal effect on the overall trend observed in the results. Subsequently, the data utilized in this research were obtained from the WOSCC and the CNKI. Given that the CNKI database predominantly encompasses Chinese-language literature and exhibits a relatively restricted scope regarding non-Chinese publications, we concurrently employed data from the WOSCC. This methodology enhances the thoroughness of our analysis and facilitates improved accuracy in international comparisons. Furthermore, the constraints associated with CiteSpace precluded this study from analyzing of author and journal co-citation on data derived from the CNKI. Nonetheless, the findings of this study provide an overview of the current states, hotspots and emerging trends in the field of SAE biomarker research, thereby offering a valuable reference for scholars engaged in future investigations.

## 5 Conclusion

Biomarkers associated with SAE possess significant research potential and hold promising prospects for clinical application. The rapid increase in the number of publications indicates that the investigation of SAE biomarkers has garnered increasing interest among researchers globally. The primary countries engaged in research are China and the United States. However, there remains a necessity to enhance collaboration and exchanges among countries and institutions. The researches include NSE, S100-β protein, inflammatory cytokines, BDNF, Aβ, TBARS, protein carbonyl content and other biomarkers, are increasingly recognized as the pivotal aspect of this research domain. The exploration of SAE biomarkers is anticipated to hold significant clinical utility in aiding the diagnosis of SAE and in evaluating the prognosis of SAE patients in the future. It is important to emphasize the necessity of focusing on both fundamental research and the translation of its findings, specifically regarding the clinical application of SAE biomarkers in the diagnosis and prognostic evaluation of SAE patients.

## Data Availability

The original contributions presented in the study are included in the article/supplementary material, further inquiries can be directed to the corresponding authors.
